# Plants in Anticancer Drug Discovery: From Molecular Mechanism to Chemoprevention

**DOI:** 10.1155/2022/5425485

**Published:** 2022-03-02

**Authors:** Arif Jamal Siddiqui, Sadaf Jahan, Ritu Singh, Juhi Saxena, Syed Amir Ashraf, Andleeb Khan, Ranjay Kumar Choudhary, Santhanaraj Balakrishnan, Riadh Badraoui, Fevzi Bardakci, Mohd Adnan

**Affiliations:** ^1^Department of Biology, College of Science, University of Hail, Hail, PO Box 2440, Saudi Arabia; ^2^Department of Medical Laboratory Sciences, College of Applied Medical Sciences, Majmaah University, Al-Majmaah 11952, Saudi Arabia; ^3^Department of Environmental Sciences, School of Earth Sciences, Central University of Rajasthan, Ajmer, India; ^4^Faculty of Applied Sciences and Biotechnology, Shoolini University of Biotechnology and Management Sciences, Bajhol, Solan, Himachal Pradesh, India; ^5^Department of Biotechnology, University Institute of Biotechnology, Chandigarh University, Gharuan, NH-95, 140413, Ludhiana-Chandigarh State Hwy, Punjab, India; ^6^Department of Clinical Nutrition, College of Applied Medical Sciences, University of Hail, Hail, PO Box 2440, Saudi Arabia; ^7^Department of Pharmacology and Toxicology, College of Pharmacy, Jazan University, Jazan 45142, Saudi Arabia; ^8^Department of Medical Equipment Technology, College of Applied Medical Sciences, Majmaah University, Al Majmaah 11952, Saudi Arabia; ^9^Department of Biomedical Engineering, Velalar College of Engineering and Technology, Erode, 638012 Tamil Nadu, India

## Abstract

Cancer is one of the primary causes of mortality globally, and the discovery of new anticancer drugs is the most important need in recent times. Natural products have been recognized as effective in fight against various diseases including cancer for over 50 years. Plants and microbes are the primary and potential sources of natural compounds to fight against cancer. Moreover, researches in the field of plant-based natural compounds have moved towards advanced and molecular level understandings from the last few decades, leading to the development of potent anticancer agents. Also, plants have been accepted as abundant and prosperous sources for the development of novel therapeutic agents for the management and prevention of different cancer types. The high toxicity of some cancer chemotherapy drugs, as well as their unfavorable side effects and drugs resistance, drives up the demand for natural compounds as new anticancer drugs. In this detailed evidence-based mechanistic review, facts and information about various medicinal plants, their bioactive compounds with their potent anticancer activities against different cancers have been gathered, with further approach to represent the molecular mechanism behind the anticancer activity of these plants. This review will be beneficial for investigators/scientists globally involved in the development of natural, safe, effective, and economical therapeutic agents/drugs against various cancers. This might be an important contribution in the field of drug discovery, where drugs can be used alone or in combination to increase the efficacy of newly synthesized drugs.

## 1. Introduction

Irrespective of advancements in oncology, cancer is yet one of the life-threatening diseases throughout the world. In 2020, approximately 19.3 million new cancer cases were reported globally, resulting in nearly 10 million deaths [1]. Despite being the localize nature of cancer, it can spread throughout the body and various organs via process of metastasis including invasion and migration [2]. From the primary location, metastasis spreads cancer cells to different locations through lymphatic or blood circulation in the body [3]. Metastasis is an intricate process comprising of multiple steps which begin with detachment, amassing, and motility of cancerous cells, resulted in attachment to endothelial cells followed with growth of cancer cells at specific sites [3, 4]. Metastasis is the leading cause for cancer-associated death, due to resistance to diverse cytotoxic agents as well as apoptosis. This is the reason of high mortality and morbidity in patients with metastatic cancer, where present chemotherapy drugs are unsuccessful in effectively eradicating the cancer cells without damaging healthy cells [5].

**Figure 1:**
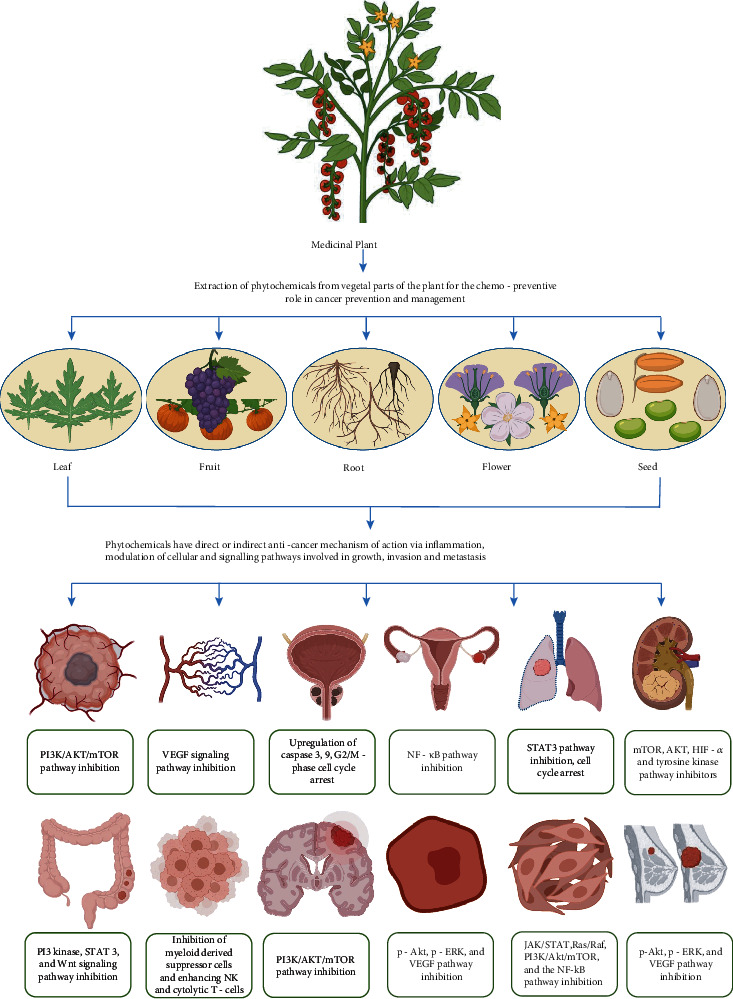
Pictorial depiction of vegetative parts of medicinal plant involved in therapeutic effects against various types of cancers and their target pathways.

Medicinal plants are a gift from nature to humans, assisting them in their quest for better health. Since prehistoric times, natural products have been recognized and used by mankind as the chief source of remedial drugs. They are still the source of excellent and potent bioactive compounds, which can directly be used as medicines [6–8]. Several plants have been found to have various kinds of biological activities, like to cure wound, skin diseases, menstrual disorders, after childbirth, fever, headache, exhalation channel infection, urethral infection, rheumatism, and cancer [9, 10]. According to current data, the plant kingdom contains nearly 250,000 plants species, of which only about 10% of medicinal plants have been studied/discovered so far for the treatment of many diseases [11, 12]. Therefore, phytochemical properties of different plants and their derived bioactive analogues occur in assorted portions of plants; like as flower, seed, stem, bark, fruit, leaf, and embryo (Figure 1) [11, 13]. Moreover, all these medicinal plants are eminent to yield numerous bioactive metabolites with various pharmacological attributes together with antidiabetic, antiosteoporotic, antimicrobial, hepatoprotective, anti-inflammatory, antimalarial, antiageing, immunomodulator, antioxidant, antihypertension, anticancer, and others (Table 1) [14–17]. Many have been shown to have anticancer properties; inhibiting cancer cell-related activating proteins, signaling pathways, and enzymes such as topoisomerase enzyme, cyclooxigenase, MMP, MAPK/ERK, TNK, Akt, cytokines, Bcl-2, PI3K, CDK4 kinases, CDK2, CDC2, mechanistic target of rapamycin (mTOR), or by activating DNA repair mechanism [11, 18, 19]. Therefore, choosing these very specific plants, which are described in detail in this review along with their mode of action and possible mechanism, and could potentially be used in the management and prevention of cancer was the primary motivation. This review is intended to open up new avenues for the various kind of treatment/therapy of cancer, focusing on certain important medicinal plants and their important bioactive compounds that have been presented to target cancer cell-activating proteins, signaling pathways, and enzymes involved in cancer progression.

## 2. Major Medicinal Plants with Anticancer Activities and Possible Mechanism

### 2.1. *Taxus chinensis* (Pilger) Rehd (*T. chinensis*)

The most common herbal medicinal component identified from *T. chinensis* is paclitaxel (PTX), a well-known first-line chemotherapy treatment/therapy for cancer disease such as ovarian and breast cancer [20]. Low bioavailability makes oral dose inoculation/administration of pure PTX as a possible anticancer drug difficult. Paclitaxel, a taxane-type diterpenoid, was discovered from the barks of *T. chinensis* and is currently this bioactive compound used as a broad-spectrum chemotherapeutic treatment/therapy against various cancer categories [21]. Numerous findings have demonstrated that PTX can cause apoptosis and cell cycle arrest by blocking microtubule depolymerization and boosting microtubule polymerization, and PTX's unusual structure and anticancer potency have piqued researchers' attention around the world [22]. The taxane extract from *T. chinensis* enhances paclitaxel bioavailability through pharmacokinetic synergy [23]. The current approach to taxol production is natural source obtained from the bark of *T. brevifolia*, which is considered as a most rich source; nonetheless, based on this method, the yield of taxol ranged from 0.001 to 0.05 percent, meaning that 8–10 kg of plant bark from 4–5 plants is required to produce one gram of pure taxol [24]. However, this supply faces issues due to its limited availability and increased vulnerability to unpredicted changes in biological and environmental conditions. Camptothecin and taxol were the first compounds with high antiproliferative activity to be studied after the National Cancer Institute program of research for novel anticancer chemicals began in 1960 [25]. NCI clinical trials have looked at taxol in the following stages of different cancers: Phase I (childhood leukemia, lymphoma, epithelial, liver, breast, and ovarian), Phase II (melanoma, head and neck, esophageal, small cell lung, colon, renal, and prostate), and Phase III (melanoma, esophageal, and melanoma) (ovarian epithelial and metastatic breast) [25–28]. Taxol was licensed by the FDA in 1992/94 as an effective treatment for breast and ovarian cancer, respectively [28]. As a result, this bioactive compound (taxol) was regarded as one of the most significant contributions to the sector of chemotherapeutics in the late twentieth century [28].

**Figure 2:**
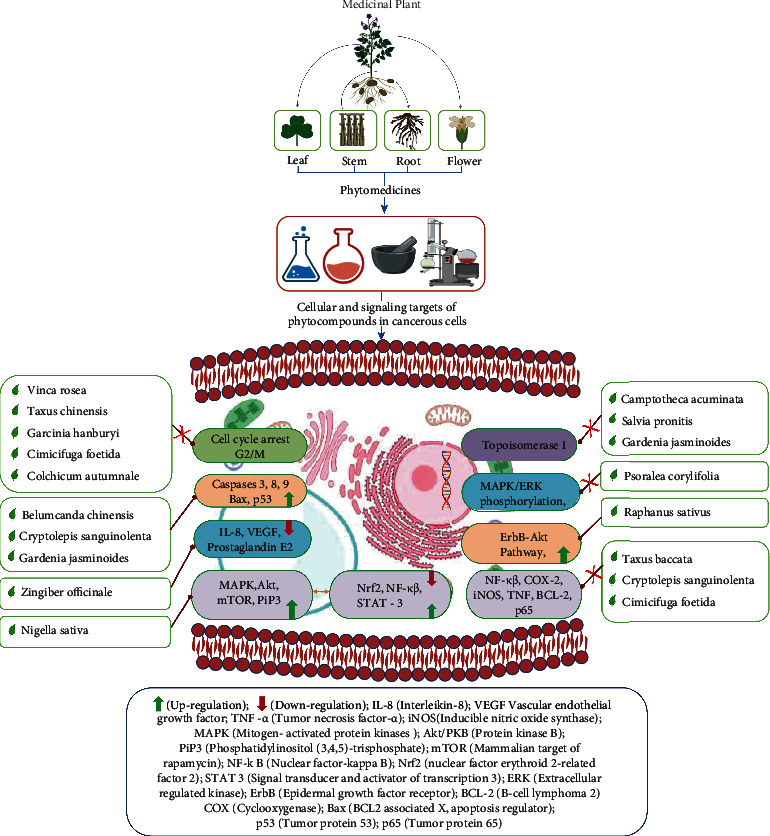
Illustrative representation of potential plants targeting specific cellular and signaling pathways depicting molecular mechanism of their possible anticancer activity.

Taxol is a highly effective anticancer treatment that works against a variety of malignancies, which include ovarian, lung, breast, head carcinomas, and AIDS-related Karposi's carcinoma [29]. Proliferation of cancer cells occurred due to abnormal cell growth and division, which causes a surge in the number of cells [29]. Tubulin is a globular protein that is located in the cytoskeleton of eukaryotic cells and plays a vital part in cancer cell mitosis [30]. During mitotic division, the dynamics of microtubules, which involves depolymerization (shrinking) and polymerization (growing), is critical for chromosome separation (Figure 2). Two various kinds of tubulins polymerize together to generate heterodimer microtubules, which are assembled to form protofilaments. Microtubules in live cells have been found to have 13 protofilaments arranged parallel to the microtubule axis [30–32]. The positive charged end of a microtubule (+tubulin) connects to the kinetochore of chromosomes, while the negative charged end (-tubulin) binds to the spindle pole. When a drug attaches/binds to tubulin, it changes the way microtubules assemble [30, 32]. Taxol is an antiproliferative medication that works in a unique way against cancer cells. It interacts with tubulin through an amino-terminal region of amino acids (that is 31 amino acid), preventing microtubule depolymerization and halting the cell cycle [31]. Microtubules organized to have 12 proto filaments rather than 13 proto filaments in the presence of taxol [32].

### 2.2. *Curcuma longa* L. (*C. longa*)

Turmeric or *C. longa*is an aromatic, nutraceutical plant. The root, a vegetal product of this plant, has been extensively utilized in Indian traditional medicine (Ayurveda) for various maladies, including parasite infection (local administration), wounds, acne, urinary tract disease, common cold, and liver disease, under various pharmaceutical formulations (systemic administration) [33, 34]. Numerous experimental studies on turmeric's therapeutic activity have revealed a wide -range of pharmacological properties, such as anti-HIV, antibacterial, antioxidant, antiangiogenic, anti-inflammatory, proapoptotic, immunomodulatory, analgesic properties with applications in Alzheimer's disease, diabetes, and arthritis [35, 36]. The main active ingredient responsible for the pharmacodynamics activity is curcumin, a polyphenol, which has displayed potent anticancer effects against numerous cancers [36]. This includes bone, breast, pancreatic, colon, lung, cervical, liver, and thymic (Figure 2) [37, 38]. Recent research strongly supports polyphenols' significance in the prevention of degenerative diseases, cancer, and cardiovascular diseases [38]. Natural polyphenols can occur in a various range of foods, including cereals, chocolates, vegetables, fruits, olive oil, and beverages like wine and tea. Curcuma aqueous extract has been shown to induce apoptosis in human colon cancer LS-174-T cells. Ozaki et al. (2000) revealed the role of curcumin in inducing apoptosis in rabbit osteoclasts as well as inhibiting bone resorption [38, 39]. Curcumin's proapoptotic effect in leukaemic Jurkat cells, COLO 205 cells, human lung carcinoma A549 cells, murine myelomonocytic leukemia WEHI-3 cells, human nasopharyngeal carcinoma cells, and NPC-TW 076 has previously been documented [38, 40]. Furthermore, curcumin is a proapoptotic agent that fights against various lymphoma cells in human [40].

### 2.3. *Zingiber officinale* Roscoe (*Z. officinale*)

*Z. officinale* (Ginger, Zingiberaceae) is a rhizomatous perennial plant used to treat digestive disorders such as dyspepsia, nausea, vomiting, gastritis, diarrhea, asthma, common cold disorders, nervous diseases, inflammation, hepatotoxicity, diabetes, migraine, hypercholesterolemia, helminthiasis, and schistosomiasis [41–43]. Since prehistoric times, ginger has been widely utilized as a spice for culinary and medicinal purposes throughout the world. Ginger has been known to be anticarcinogenic through numerous routes and to have chemo-preventive activity in colon cancer [43]. Human colorectal cancer cells were also suppressed by gingerol (phytochemical found in ginger). Efficacy of ginger has been tested on developed tumor in mice in one study and showed potential reduction in tumor size [44]. In mice, the effects of continuous therapy with ginger rhizome hot water extract on spontaneous mammary cancer were studied. The formation of mammary tumors was dramatically prevented in the mice given free access to ginger extract (0.125 percent) in drinking water [44–46]. Gingerol also induces auto phagocytosis and apoptosis, which destroys ovarian cancer cells (self-digestion). The presence of a proinflammatory state is hypothesized to participate in the enlargement of ovarian cancer. A number of critical inflammatory markers (interleukin-8, vascular endothelial growth factor, and prostaglandin E2) found to be reduced in ovarian cancer cells with treatment of ginger [46]. The antiproliferative and antioxidant activities of the methanol extract of cannibal rhizome on human cervical HeLa cancer cells and human breast cancer MDA-MB-231 cancer cells were also studied [46].

### 2.4. *Camptotheca acuminata* Decne (*C. acuminata*)

This medicinal plant belong to Nyssaceae family and basically was found in Tibet and southern China [47]. *C. acuminata* is well know for broad-range of biological activities such as anticancer, antivirus, antipsoriatic, antifungal, anti-inflammation, antibacterial, and antiparasitic [48–50]. Furthermore, *C. acuminata* contains various natural active compounds, like alkaloids, flavonoids, and glycosides [50]. It contains alkaloid camptothecin (CPT) and betulinic acid (BA), which can be isolated from stem, bark, and fruit of *C. acuminata* plant [51]. The anticancer mechanism of action of CPT is by inhibiting topoisomerase 1. CPT binds to a complex, which contain deoxyribose nucleic acid (DNA) and topoisomerase I, hence, inhibiting the reassembly of the DNA strands of a single chain [51, 52]. When the CPT is combined with the DNA–topoisomerase complex, the bonds at the nick sites are not restored, and the double DNA chain structure is damaged [52]. The DNA damage in tumor cells treated with CPT and its derivatives is most likely caused by double-strand breaks introduced by the replication process [25, 52]. Therefore, CPT also has been engaged for the treatment of ovarian and lung cancer [53]. On the other hand, BA is involved in treatment of cancer, HIV, and bacterial diseases.

### 2.5. *Vinca rosea* L. (*Catharanthus roseus* (L.) G. Don)

This medicinal and ornamental plant is a member of Apocynaceae family and commonly known as pink periwinkle or Madagascar periwinkle [54]. *V. rosea* is rich in alkaloids with wide-ranging biological activities and use in treatment of several types of cancers, such as lung, breast, leukemia, acute lymphoblastic leukemia, testicular germ cell tumor, Kaposi sarcoma, Hodgkin's lymphoma follicular lymphoma, ovarian germ cell tumor, retinoblastoma, Ewing sarcoma, and rhabdomyosarcoma [54, 55]. Furthermore, *V. rosea* contains Vinca alkaloids: that are vindesine, vincristine, vinorelbine, and vinblastine [56, 57]. All these Vinca alkaloids were the first natural compounds to go into clinical trial against several cancers and have been endorsed and licensed by Food and Drug Administration (FDA) [58]. It has been known that usage of these alkaloids in low doses interferes with the microtubular activity, while at high doses, causing cell cycle arrest and apoptosis [58, 59]. These alkaloids are currently in use for the treatment of various cancers.

### 2.6. *Belamcanda chinensis* L. (*B. chinensis*)

*B. chinensis*is a traditional Chinese medicinal plant, belongs to the Iridaceae family and commonly found in northeast Asia region [60, 61]. The biological activity of this plant include antitumor, antioxidant, antidiabetic, estrogen-like, hepatoprotective, antibacterial, and anti-inflammatory [61]. It contains several bioactive compounds like flavonoids, terpenoids, organic acids, and quinones [62]. The flavonoids and terpenoids are mostly used in the treatment of cancer [63]. Terpenoids (pentacyclic triterpenoids) are also one class of natural compounds introduced in clinical trials [63]. However, ursolic acid, betulone, and betulonic acid are isolated from root of *B. chinensis* medicinal plant and mostly all these compounds are utilized in treatment/therapy of numerous type of cancers, such as solid tumor, breast cancer, liver cancer, prostrate carcinoma, gastric carcinoma, and T-cell leukemia [64, 65]. However, against different types of cancer cells, betulonic acid has shown most significant antitumor activity at 20 *μ*mol/L: for example, prostatic cancer cell line PC3 (rate of inhibition = 52%), breast cancer cell line MCF-7 (rate of inhibition = 56%), and human gastric cancer cell line MGC-803 (rate of inhibition = 68%) [63]. Furthermore, the ability of betulonic acid to follow apoptotic pathways through the mitochondrial signaling cascade, which implies the expression of caspases 3 and 9, as well as the proteins p53 and Bax, is one of its antitumor properties [63]. Therefore, this is most promising agent which can be used for the cancer chemoprevention or management.

### 2.7. *Cryptolepis sanguinolenta* (Lindl.) Schlechter (*C. sanguinolenta*)

*C. sanguinolenta* is an African scrambling thin-stemmed shrub. It has traditionally been used in West Africa to treat the diarrhea, malaria, and respiratory problems [66]. Furthermore, different synthetic derivatives of specific alkaloidal isolates from *C. sanguinolenta* have also been studied for the anticancer action. *C. sanguinolenta* has been mostly linked to its principal alkaloid, cryptolepine [66, 67], which has demonstrated to block the NF-*κ*B activity in a variety of cells [68]. Along with that, in human lung adenocarcinoma A549 cells, cryptolepine stimulates cell cycle arrest and apoptosis [66, 68]. Cytotoxic and anti-inflammatory effects are mediated by interfering with NF-*κ*B activity, which results in reduction of inflammatory as well as antiapoptotic genes such iNOS, TNF-*α*, COX-2, and Bcl-2 [68]. The proapoptotic genes p53, p21, Bax, caspase, and cytochrome c are all upregulated when NF-*κ*B is inhibited [69].

Olajide et al. 2013 studied that apoptotic-inducing impact of cryptolepine is mediated through the NF-*κ*B signaling pathway during different study [69]. It was observed that, after 24 hours of therapy, cryptolepine decreased A549 cell growth dose-dependently with upregulation of caspase-3 [69]. Cryptolepine reduced TNF-induced IB phosphorylation and NF-*κ*Bp65 nuclear translocation, according to protein analysis [67, 69]. Moreover, significant DNA damage is caused by cryptolepine by blocking the topoisomerase I and II activity [68, 70]. This damage led in increased phosphorylation of BRCA1, Chk1/Chk2, H2AX, and ATM/ATR, as well as p53 signaling cascade activation, which included increased protein expression of the cyclin-dependent kinases p21 and p16 [71]. In human nonmelanoma skin cancer cells, these cryptolepine-induced alterations resulted in a considerable reduction in cell viability and colony formation, as well as an expend in apoptotic cell death [71].

### 2.8. *Garcinia hanburyi* Hook (*G. hanburyi*)

Garcinia is a Clusiaceae genus found in Africa, New Caledonia, Southeast Asia, Brazil, and Polynesia [72]. Garcinia plants contain a wide range of biologically active metabolites that have gotten a lot of interest in recent decades because of the chemical compositions of their extracts, which contain chemicals that have been demonstrated to have positive effects in a variety of ailments [72]. There are several species found, but the following five are the best studied and have numerous therapeutic properties: *G. mangstana*, *G. pedunculata*, *G. gardneriana*, *G. brasiliensis*, and *G. cambogia*. These species are known to have potent anti-inflammatory effects; such as pain, wounds, treatment for skin diseases and infections, with analgesic, antioxidant, antitumor, antifungal, anticancer, and anti-inflammatory properties [73, 74]. They are a rich and natural source of biologically active compounds with various other therapeutic properties Moreover, they also contain amine, with antiulcer, antibacterial, antiviral, vasodilator, hypolipidemic, and hepatoprotective properties [74]. Moreover, *G. hanburyi* has anticancer effects in different kinds of human cancer cells, such as gastric carcinoma, hepatocellular carcinoma, breast cancer, prostate cancer, and lung cancer [75].

Garcinia is rich in polyisoprenylated benzene derivatives (polyphenols, benzophenones, xanthones, and bioflavonoids) [76]. Garcinia-derived biflavonoids have also been studied for a variety of functions, including chemoprevention [77]. Kolaviron (extract from the seed of Gracinia) is believed to have the capability to eliminate free radicals, inhibit stress-related proteins and interfere with the DNA binding activity of certain transcription factors. Thus, it helps as anticancer agent [77, 78]. However, forbesione, a caged xanthone derived from *G. hanburyi*, has been shown in human CCA cell lines to reduce growth and cause apoptosis [77]. In addition, it was found that the expression of p27 and p21 was increased, which may describe why proliferating bile duct cell markers and cell nuclear antigen in forbesone-treated Ham-1 cells *in vitro* and in forbesone-treated hamster tumor tissues Cytokeratin 19 are reduced. In addition, forbesione promotes apoptosis through a variety of mechanisms [79]. Increased expression of Fas, Fas-related death domains, and activated caspase-3, as well as decreased expression of procaspase-3 and 8, activates the death receptor pathway [79, 80]. Increased expression of activated caspase-9, B-cell lymphoma- (Bcl-) 2-like protein 4 and B-inhibitors, expression of Bcl-2, survivin, procaspase-9, and nuclear factor-B decreased p65, driving mitochondria way [80]. Lower expression of procaspase-12 and higher expression of activated caspase-12 stimulated the endoplasmic reticulum pathway. In forbesione-treated hamsters, no adverse effects or toxicity were identified. As a result, forbesione is observed as a promising cancer treatment candidate that warrants additional research [79, 80].

### 2.9. *Psoralea corylifolia* L. (Buguchi) (*P. corylifolia*)

This medicinal plant is member of Leguminosae family and was used in ancient times for the treatment of several ailments and is originally distributed in Asian regions [81]. It is known forwide-range of biological activtiessuch as anticancer properties, anti-inflammatory, antibacterial, antidepressant, antioxidant, and antipsoriatic [81]. Furthermore, this medicinal plant encompasses numerous bioactive components like as psoralidin, meroterpenes, flavonoids, and coumarins. Furthermore, bioactive compounds psoracoumestan and arylcoumarin from *P. corylifolia* displayed robust anticancer potential by strongly blocking MAPK/ERK kinase phosphorylation enzyme system [82, 83]. Another bioactive compound psoralidin has found to be potentially important in treatment of cancer. Psoralidin is involved in different types of cancers like breast, liver, and others [82, 84]. In a study, psoralidin an estrogen receptors agonist was found to induce pS2 gene activity in MCF7 breast cancer cells with an EC_50_ value of ERE-reporter gene transcription activity of 1.85 *μ*M [82, 85]. While, seed extract of *P. corylifolia*induced apoptosis in the human breast cancer (MCF-7) cells followed by mitochondrial cell death [86]. Another study stated that combination of psoralidin and neobavaisoflavone with TNF-related apoptosis-inducing ligand (TRAIL) has highlighted their anticancer activity through inducing apoptosis in human adenocarcinoma prostate cancer cells [84, 87]. Another study clearly revealed that psoralidin combination with TRAIL influences apoptosis in HeLa cells by upregulating the expression of death receptor [84, 87]. Furthermore, in the same study, it has been showed that psoralidin bioactive compound have anticancer activity against human lung cancer cell by using different extracts of *P. corylifolia* [84, 87].

### 2.10. *Cimicifuga foetida* L. (*C. foetida*)

This medicinal plant is a member of Ranunculaceae family and originally was found in Asian region such as China, India, and Tibet [88]. This medicinal plant has been used a traditional Chinese herbal medicine globally since old times and commonly known as Shengma [88]. *C. foetida* is mostly used in the treatment/cure of headache, sore throat, aphtha, uterine, prolapse, archoptosis, spot poison, and nonerupting measles and many other related diseases [88, 89]. *C. foetida* has wide-ranging biological activities comprising like as antitumor, anti-inflammatory, antiviral, antimenopause, analgesic, antiosteoporosis, and antipyretic [88, 89]. Its potent bioactive compounds are phenylpropanoids, lignans, amides, chromones, cycloartane triterpenoids, and other compounds, which are extracted from the rhizome of *C. foetida* [90]. Therefore, phenolic compounds and triterpenoids have been shown effective anticancer activities in both *in-vitro* and *in-vivo* studies [91, 92]. Furthermore, *C. foetida* is often used as estrogen-based replacement therapy to get rid of menopausal symptom and detoxification. Triterpenoids in *C. foetida* synergistically impedes the proliferation of hepatocellular cells, breast cancer cell line, and prostate cancer by triggering cell cycle G2/M arrest and apoptosis [93]. Moreover, triterpenoid (KHF16) compounds have the ability to induce cell cycle G2/M phase arrest and apoptosis in different cell lines, as well as potential to block TNF*α*-induced p65 nuclear translocation, IKK*α*/*β* phosphorylation in ER*α*/PR/HER2 triple-negative breast cancer cells [93]. Hence, bioactive compounds from *C. foetida* have great potential to inhibit several cancers and must undergo for clinical trials.

### 2.11. *Taxus baccata* L. (*T. baccata*)

This plant is a member of Taxaceae family and generally known as English yew or European yew. It is widely found in Eastern Asia, Europe, and North America [94, 95]. *T. baccata* is mostly used for ornamental landscaping and inflammatory diseases, due to the occurence of lignin derivatives. *T. baccata* contains large amount of bioactive compounds such as Paclitaxel (taxol), taxusin, baccatin, taxoids viz., baccatin, taxine, lignans, phenols, phenolic glucoside, sugar derivatives, steroids, and flavonoids (3-O-rutinoside quercetin, 3-O-rutinoside myricetin, and quercetin) [96, 97]. *T. baccata* possesses a wide range of biological activities; anticancer, antimalarial, antirheumatic, abortifacient, anti-inflammatory, antinociceptive, and septic shock activities [96, 97]. However, two bioactive compounds of *T. baccata*, lariciresinol and isolariciresinol, have been reported for anticancer activities [98]. Lariciresinol and isolariciresinol are lignans, which were revealed to have effective inhibitory effect on tumor necrosis factor (TNF) *in vitro* [98]. It is known that TNF is one of the main Th1 cytokine released during initial phase of chronic, and acute inflammatory diseases includes rheumatoid arthritis, asthma, and septic shock [99]. Therefore, paclitaxel (taxol) and taxanes isolated from *T. baccata* have been permitted for therapeutics of numerous cancer-related diseases such as lung cancer, Kaposi's sarcoma, ovarian cancer, and breast cancer [28, 96]. Currently, paclitaxel and taxanes are under clinical trial for the treatment of other cancers in combination with other bioactive compounds/chemotherapeutic medications [96]. Furthermore, taxanes have potential to inhibit the mitosis division.

### 2.12. Viscum album L. (V. album)

This plant (*V. album*) is a hemi-parasitic, evergreen shrub plant is a member of Santalaceae family and commonly known as Mistletoe or European mistletoe [100]. It is widely distributed in Europe, southwest and northwest Africa, and central Asia. Several bioactive compounds are known to be present in *V. album* such as flavonoids, phenylpropanoids, alkaloids, proteins, carbohydrates, oligosaccharides, polysaccharides, triterpenes, steroids, lipophilic molecules, viscumneoside XII, XIII, XIV, lectins, and conjugated acetylene compounds [100–102]. Viscotoxins are thionines and are classified as alpha and beta is the major low molecular protein of *V. album*, which is extensively reported [103]. The biological activities of *V. album* have been reported to be anticancer, antidiabetic, antioxidant, anti-inflammatory, sedative, antihypertensive, and hepatoprotective [101]. Furthermore, *V. album* has been used mostly to treat high blood pressure, arthrosis, hemorrhages, arteriosclerosis, diabetes, menstrual disturbances, migraines, epilepsy, endometriosis, asthma bronchiale, and neuralgias in the last 200 years [104, 105]. Several preclinical studies have been reported that *V. album* showed proapoptotic, immunostimulatory, and cytotoxic effects [106]. Furthermore, in mouse/animal models, *V. album* extract has showed direct antitumour activity, while indirect activity showed via initiation of various immune system pathways [106]. The main phytochemicals of *V. album* such as lectins and viscotoxins are described to have anticancer activity [107]. Lectins and viscotoxins play an important role in cancer treatment because of their cytotoxic and apoptotic effects. They are further able to increase cytokine secretion, stimulate immune cells phagocytosis, induce macrophage cytotoxicity, and enhance *in vitro* cytotoxic effects on various cell lines [107, 108]. Efficacy of *V. album* on the activity of natural killer (NK) cells and T-cytotoxic cells was also investigated in another study [108]. Although inhibition of NK cells cytotoxic activity was also discovered in some studies, the majority of them showed an increase in cell concentration and improved function [109, 110]. Whereas, several studies confirmed the stability of tumor, reduction in tumor growth, or reduction in metastases with improved survival rate [108, 109, 111]. Hence, *V. album* is an excellent example of medicinal plant that acts as a link between conventional and alternative medicine.

### 2.13. *Gardenia jasminoides* J. Ellis (*G. jasminoides*)

*G. jasminoides* belongs to Rubiaceae family and mostly found in Asian reason such as China, India, Korea, Nepal, Tibet, and Bhutan. It is mostly used in the treatment of hypertension, edema, inflammation, fever, headache, jaundice, and hepatic disorders [112, 113]. *G. jasminoides* possesses several biological activities; anticancer, antidepression, antioxidant, hypoglycemic effect, antidiabetic, anti-inflammatory, improvement in the quality of sleep, antigastritic, antiarthritis, antihyperlipidemia, and also inhibition of retinal damage [113, 114]. *G. jasminoides* is also widely used as a natural yellow dye and as a traditional Chinese medicine since ancient times. *G. jasminoides* contains many bioactive compounds such as geniposide, crocin, genipin, gardenoside, and iridiod [113]. However, chemical components of this medicinal plant have been isolated and characterized as/including volatile compounds, glucosides, triterpenoids, organic acids, and iridoid [114]. Aliphatic acids, esters, alcohols, ketones, aldehydes, and aromatic derivatives are the main volatile compounds found in *G. jasminoides* essential oil [106]. Furthermore, *G. jasminoides* fruit contains iridoid glycosides and crocin as main bioactive compounds with a potential to exhibit antitumor, antioxidant, cytotoxic, and antihyperlipidemic effects [115, 116]. *G. jasminoides* extract can inhibit the activity of topoisomerase 1, which seemingly encourages the formation of supercoiled DNA [116, 117]. Lim et al. (2010) reported that on KB oral cancer cells, the cytotoxic effect of *G. jasminoides* extract dichloromethane fraction increased in a dose-dependent manner [117]. This cytotoxicity was not seen in the normal human epidermal keratinocyte HaCaT cell line, but was found to be effective against oral cancer KB cell line. Furthermore, Lim et al. (2010) demonstrated that the dichloromethane fraction of *G. jasminoides* extract induced apoptosis by increasing the activities of caspase-3, 8, and 9, as well as the cleavage of poly (ADP-ribose) polymerase [117]. Hence, these novel findings suggest that *G. jasminoides* extract could be a candidate for the development of novel anticancer drug.

### 2.14. *Colchicum autumnal* L. (*C. autumnale*)

*C. autumnale*, commonly known as autumn crocus, is a flowering plant which belongs to the family of Colchicaceae. It is native to Great Britain and Ireland. *C. autumnale* is considered as a toxic plant because of the presence of colchinine, which is found in the bulb like corms of the plant [118]. Colchicine is the main alkaloid of *C. autumnale*. It has narrow therapeutic index and is used effectively as a remedy against gout, Behçet's disease, and familial Mediterranean fever at many places [119]. The pain-relieving, anti-inflammatory, and antiproliferative effects of colchicine are closely linked with its ability to bind with tubulin, which plays an important role in cell division. Colchicine blocks the cell cycle at the G2/M phase inhibiting the action of tubulin, thereby the formation of microtubule severely damages the internal scaffolding of the cells and trigger apoptosis [120]. Owing to its high toxicity, colchicine has not found significant use in cancer treatment; however, it is still used as a lead compound for the generation of potential anticancer drugs [121].

### 2.15. *Salvia pronitis* Hance (*S. prionitis*)

*S. prionitis* is an annual herb, which belongs to the family of Lamiaceae. It is native to the southern part of mainland China. A diterpenoid quinone named as Saprorthoquinone is the main compound isolated from *S. prionitis* and is used as remedy of fever, tonsillitis, pneumonia, and diarrhea [122, 123]. In *in vivo* xenograft models of LAX-83 lung adenocarcinoma cells, A-549 lung, Lewis lung, and S-180 sarcoma, Salvicine, which is a diterpenoid quinone from *S. prionitis*, displayed significant growth inhibitory activity [124, 125]. It also bears cytotoxic effect on multidrug-resistant (MDR) tumor cells [126]. Salvicine exerts its antineoplastic effects and induces apoptosis by acting as a nonintercalative topoisomerase II inhibitor. Additionally, Salvicine increases the level of intracellular H_2_O_2_ and is also linked with the occurrence of DNS double-strand breaks [127]. Salvicine directly reacts with GSH and results in significant depletion of intracellular GSH. It has been suggested that both GSH-depletion-driven H_2_O_2_ generation and Topo II inhibition are critical for Salvicine-mediated DNA double-strand breaks and apoptosis [127].

### 2.16. *Raphanus sativus* L. (*R. sativus*)

*R. sativus* is an important and traditional annual vegetable in many countries, which belongs to the family Cruciferae. The extract of *R. sativus* is known to regulate phase I and II detoxification system inhibiting the proliferation of HepG2 cells, significantly [128]. Its extract contains glucosinolate compounds such as 4-methylthio-3-butenyl isothiocyanate and glucoraphasatin, which imparts anticancer property to *R. sativus* [129]. The extracts of *R. sativus* exhibit effective cytotoxicity against HCT116 colon cancer cell line by inducing apoptosis [130]. Similarly, in the MDA-MB-231 breast cancer cell line, the aerial extract showed effective cytotoxicity via the ErbB-Akt pathway [131].

### 2.17. *Tinospora cordifolia* (Willd.) Miers (*T. cordifolia*)

*T. cordifolia* is an herbaceous vine and belongs to the family of Menispermaceae. It is indigenous to the tropical regions of the Indian subcontinent. It has been used in the Indian Ayurvedic medicines for centuries to treat jaundice, diabetes, and rheumatoid arthritis and is also used as an immunostimulant. *T. cordifolia* is known to have antineoplastic, antioxidant, hepatoprotective, hypolipidemic, and immunologic properties [132]. It is reported to contain a wide array of biologically active compounds which are isolated from the different parts of its body. The main bioactive compounds having therapeutic values include diterpenoid, polysaccharides, lactones, aliphatic compounds, steroids, alkaloids, sesquiterpenoid, and glycosides [133, 134]. The extract of *T. cordifolia* is extensively used in medicinal formulation for its antiperiodic, antispasmodic, antimicrobial, antiosteoporotic, anti-inflammatory, antiarthritic, antiallergic, and antidiabetic properties [135, 136]. *T. cordifolia* is reported to overcome cyclophosphamide (CP) induced toxicities in cancer. In diethylnitrosamine induced hepatocellular carcinoma model in rats, *T. cordifolia* has been reported for its chemo-preventive potential. The chemo-preventive action of *T. cordifolia* is manifested through the decrease in antioxidant activities via superoxide dismutase, catalase, and detoxification enzymes like GPx and GSH, however, increase in the hepatic marker's activities such as lactate dehydrogenase, serum glutamic pyruvate transaminase, serum glutamic oxaloacetic transaminase, and decreased serum transaminase level [136, 137].

### 2.18. *Nigella sativa* (N. sativa)

This medicinal plant is a member of Ranunculaceae family with annual flowering routine and is native to Eastern Europe and Western Asia. The main bioactive compound which confers anticancer properties to *N. sativa* is Thymoquinone (2-methyl-5-isopropyl-1, 4-benzoquinone). Thymoquinone exhibits cell death of abnormal cells with growth inhibitory activities. According to one study, it is reported that Thymoquinone blocked the tumor growth in murine models [138]. It has been reported that in breast cancer patients, severity of acute radiation dermatitis is reduced after the topical application of *N. sativa* gel [139]. Furthermore, in children with brain tumors, reduction in febrile neutropenia has been reported after taking the *N. sativa* seeds orally [140]. Thymoquinone affects the triggering of numerous upregulation of tyrosine kinases (e.g., PIP3, mTOR, Akt, and MAPK) as well as phosphorylation process of signaling pathways, which are participated in tumor cell proliferation [141, 142]. Thymoquinone also regulates the activation of transcriptional factors (e.g., NF-*κ*B, Nrf2, STAT-3, NF-*κ*B, and Nrf2) counteracting diverse tumorigenic developments, such as cell survival, cell proliferation, cell invasion, inflammation, angiogenesis, and metastasis [142–144]. Thymoquinone shows chemo-preventive properties by attenuating the production of proinflammatory mediators (e.g., prostaglandins, chemokines, and cytokines), upregulating the cytoprotective enzymes (e.g., oxidoreductase, superoxide dismutase, and glutathione S-transferase) and downregulating the carcinogen metabolizing enzymes (e.g., CYP 3A4 and CYP 1A2) [142]. Thymoquinone is also reported to sensitize cancer cells to conventional chemotherapy, immunotherapy, and radiotherapy by modulating the resistance mechanisms [145, 146]. Though thymoquinone bears great anticancer properties, its lower efficacy and poor bioavailability are the major constraints as primary therapeutic agent against cancer [147–149].

## 3. Future Prospective and Conclusion

Medicinal plant-based natural bioactive compounds have played a significant role in human health. As details disputed in this present review, focus was all given in developing new anticancer agents or new treatment strategies protocol against various cancers. As a result, several natural bioactive compounds/metabolites can be investigated/explored in order to explore the mechanistic action and detailed structure types for developing novel anticancer drugs. Though, the drug discovery process, which includes compound isolation, characterization, biological activity determination, preclinical, and clinical trials, is a lengthy and costly process but green alternatives for cancer therapy without any after effects are worth any cost. Current developments in modern techniques and advance instrumentations helped in the identification of several new and more effective novel bioactive drugs obtained from potent medicinal plants, which may further lead in developing effective anticancer compounds. Furthermore, due to their therapeutic potential, they can not only be looked as potential drug against cancer but also promising and effective supplementary foods or nutraceuticals, which are helpful in promoting good health as well as in the management of cancer. Plants are the rich source of bioactive compounds, with active molecules that can act on a variety of biochemical/signaling pathways. Recent researches explore the novelty and effectiveness of anticancer therapeutic drugs derived from plants. Furthermore, some of the medicinal plants and their bioactive compounds have been found to have significant effect against various cancers, primarily breast cancer, lung cancer, leukemia, Kaposi sarcoma, testicular germ cell tumor, Hodgkin's lymphoma, follicular lymphoma, ovarian germ cell tumor, acute lymphoblastic leukemia, acute lymphoblastic leukemia, and others with possible mechanism of action revealed. Hence, it is critical that their identification/discovery be pursued further in order to provide the public with more efficient and compliant therapeutic agents against cancer. The ultimate basis of this review is to reveal the potency of plants as anticancer agents and concluding that most of the research in this field always ends up in early stages without proceeding to identify the full potential of these plants and their therapeutic significance at molecular level, with only a few under *in vivo* experiments and clinical trials as well. Despite of the various potent advantages, there are some concerns regarding the safety and toxicity of medicinal and herbal plants. Some of the medicinal plants as whole can be allergenic or harmful for sensitive populations, while in other cases, certain parts of a plant may be edible and another part may be poisonous. Therefore, toxicological assessment of any medicinal or herbal plant is mandatorily required to identify the adverse effects and to determine limits of exposure level at which such effects occur. Underestimating the toxicological challenges associated with the use of drugs derived from medicinal plants may pose serious health concerns.

## Figures and Tables

**Table 1:** List of plants and their potent bioactive phytocompounds for the possible therapeutic use in prevention and management of various cancers.

Vegetal part and botanical name	Picture	Bioactive phytocompounds	Biological functions	Therapeutic effect against various cancers	References
*Taxus chinensis* (Pilger) Rehd (bark)	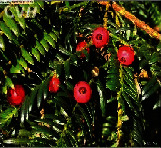	Paclitaxel (taxol)	Anticancer, antioxidant, and antiageing	Breast, liver, ovarian, colon, lung and esophageal cancer, lymphoma, childhood leukemia, melanoma	[20–22, 25, 26, 150]
*Curcuma longa* Linnaeus (rhizome)	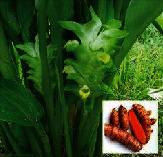	Curcumin, demethoxycurcumin, bisdemethoxycurcumin, germacrone, furanodienone, zederone, and ar-turmerone	Anticancer, antiangiogenic, antioxidant, anti-inflammatory, anti-HIV, antibacterial, and immunomodulatory	Colon, cervical, lung, thymic, brain, pancreatic, breast, bone cancers, and liver cancer	[34–37, 151]
*Zingiber officinale* roscoe (rhizome)	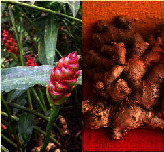	Phenolic and terpene	Anticancer, anti-inflammatory, antioxidant, immunomodulatory, antiangiogenic, and antibacterial	Colon cancer, ovarian cancer, and breast cancer	[41–43, 46, 152]
*Camptotheca acuminata* Decne (leaf, flower, stem, fruit, root)	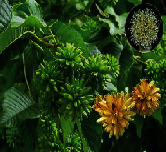	Alkaloids, flavonoids, and glycosides	Anticancer, antivirus, antipsoriatic, antifungal, anti-inflammation, antibacterial, and antiparasitic	Lung and ovarian cancer	[48–50, 153]
*Vinca rosea* L. (*Catharanthus roseus* (L.) G. Don) (leaf)	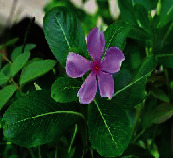	Vinca alkaloids: vindesine, vincristine, vinorelbine, and vinblastine	Anticancer, antioxidant, anti-inflammatory	Lung cancer, breast cancer, Hodgkin's lymphoma, leukemia, Kaposi sarcoma, Ewing sarcoma, follicular lymphoma, retinoblastoma, ovarian germ cell tumor, acute lymphoblastic leukemia rhabdomyosarcoma, testicular germ cell tumor	[54–56, 154]
*Belamcanda chinensis* L. DC. (root)	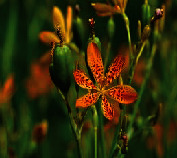	Flavonoids, terpenoids, organic acids, and quinones	Antitumor, antioxidant, antibacterial, antidiabetic, estrogen-like, hepatoprotective, and anti-inflammatory	Breast cancer, liver cancer, prostrate carcinoma, gastric carcinoma, T-cell leukemia	[61–63, 65, 155]
*Cryptolepis sanguinolenta* (Lindl.) Schltr. (leaf and root)	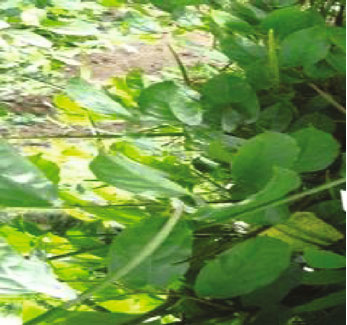	Alkaloids, flavones, and tannin	Anticancer, antibacterial, antiparasitic, anti-inflammatory, antifungal, antidiabetic, and antioxidant	Lung cancer	[66, 67, 156]
*Garcinia hanburryi* hook (fruit, leaf, and seed)	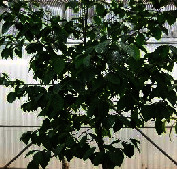	Polyphenols, benzophenones, xanthones, and bioflavonoids	Anticancer, anti-inflammatory, antioxidant, antitumor, antifungal, antiulcer, antibacterial, antiviral.	Breast cancer, lung cancer, hepatocellular carcinoma, prostate cancer, and gastric carcinoma.	[74–77, 157]
*Psoralea corylifolia* L. (Buguchi) (whole plant)	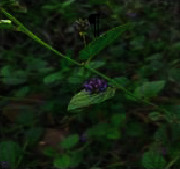	Psoralidin, meroterpenes, coumarins, and flavonoids	Anticancer, anti-inflammatory, antibacterial, antidepressant, antioxidant, and antipsoriatic.	Breast, prostate, and lung cancer	[81, 82, 84, 158]
*Cimicifuga foetida* L. (rhizome)	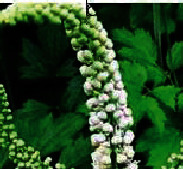	Phenylpropanoids, lignans, cycloartane triterpenoids, chromones, amides	Antitumor, anti-inflammatory, antiviral, antimenopause, analgesic, antiosteoporosis, and antipyretic	Breast, prostate, and liver cancer	[88, 89, 93, 159]
*Taxus baccata* L. (leaf and bark)	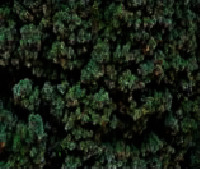	Paclitaxel (taxol), taxusin, baccatin, taxoids viz., baccatin, taxine, lignans, phenols steroids, flavonoids	Anticancer, antimalarial, antirheumatic, abortifacient, anti-inflammatory antinociceptive, and septic shock	Kaposi's sarcoma, breast, ovarian, and lung cancer	[94, 96, 97, 160]
*Viscum Album* L. (stem, leaf, and fruit)	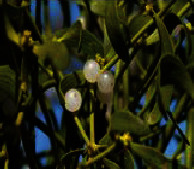	Flavonoids, phenylpropanoids, alkaloids, proteins, triterpenes, steroids, lipophilic molecules, viscumneoside XII, XIII, XIV, lectins, and conjugated acetylene	Anticancer, antidiabetic, antioxidant, anti-inflammatory, sedative, antihypertensive, and hepatoprotective	Breast cancer and gynecological cancer	[101, 102, 109, 111, 161]
*Gardenia jasminoides* J. Ellis (stem, bark, and fruit)	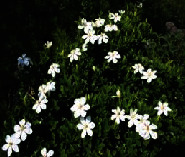	Geniposide, crocin, genipin, gardenoside, and iridiod	Anticancer, antidepression activity, antioxidant, hypoglycemic effect, antidiabetic, anti-inflammatory, improvement in the quality of sleep, antigastritis, antiarthritis, antihyperlipidemia	Brain tumor, oral cancer, liver cancer	[113, 114, 116, 162, 163]
*Colchicum autumnale* L. (bulb, flowers, and leave)	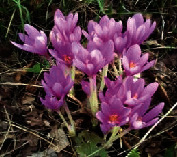	Colchicine	Anticancer, anti-inflammatory, and antiproliferative	Solid tumor, leukemia	[118, 119, 121, 164]
*Salvia prionitis* Hance (root)	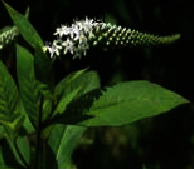	Diterpenoid quinone, salvicine	Anticancer, antitumor, antimicrobial	Lung cancer and solid tumor	[122, 124, 127]
*Raphanus sativus* L. (root, stem, leaf)	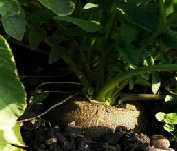	Flavanoid, glucosinolates, folic acid, flavonoids, polyphenolics, dietary fiber, vitamin A and C	Anticancer, antimicrobial, and anti-inflammatory	Colon cancer and breast cancer	[128–131, 165]
*Tinospora cordifolia* (Willd.) Miers (bark, leaf, flower, and stem)	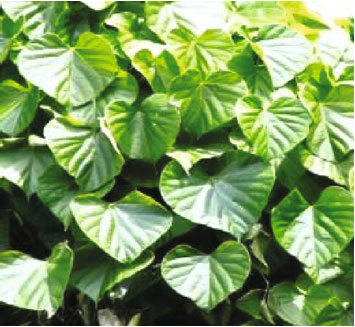	Polysaccharides, aliphatic compounds, phenolics, sesquiterpenoid, steroids, diterpenoid lactones, alkaloids, glycosides	Anticancer, antineoplastic, antioxidant, hepatoprotective, hypolipidemic, antiperiodic, antispasmodic, anti-inflammatory, antimicrobial, antiosteoporotic, antidiabetic, antiarthritic, antiallergic, and immunologic	Breast cancer and tumor	[132–137, 166]
*Nigella sativa* L. (seed)	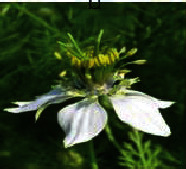	Thymoquinone, dithymoquinone, and dihydrothymoquinone	Anti-inflammatory, anticancer, antioxidant, antibacterial, antiangiogenic, antidiabetic, and organ-protective	Breast cancer and tumor	[138–142, 167]
